# Sociocultural correlates of eating pathology in college women from US and Iran

**DOI:** 10.3389/fpsyg.2022.966810

**Published:** 2022-09-28

**Authors:** Reza N. Sahlan, Liya M. Akoury, Jessica Habashy, Kristen M. Culbert, Cortney S. Warren

**Affiliations:** ^1^Department of Clinical Psychology, Iran University of Medical Sciences, Tehran, Iran; ^2^Clinical Psychologist, Aviva Psychology Services, Boston, MA, United States; ^3^Department of Psychology, University of Nevada, Las Vegas, NV, United States; ^4^Department of Psychology, Michigan State University, East Lansing, MI, United States; ^5^Choose Honesty, LLC, Chicago, IL, United States

**Keywords:** sociocultural influences, eating pathology, college women, US, Iran

## Abstract

**Objective:**

The buffering role of the hijab as a protective factor against eating pathology has been questioned in countries where wearing the hijab is compulsory, such as Iran; and, cross-cultural comparisons of body image in Iranian and Western women are sparse. Consequently, we examined sociocultural correlates of eating pathology in US and Iranian women.

**Method:**

College women from the US (*n* = 709) and Iran (*n* = 331) completed the Eating Disorder Examination-Questionnaire (EDE-Q) and the Sociocultural Attitude Toward to Appearance Questionnaire-4 (SATAQ-4). Prior to examining main hypotheses, we evaluated whether the scales perform similarly (i.e., establish measurement invariance) by culture.

**Results:**

The EDE-Q and SATAQ-4 were not invariant by culture indicating that the scales performed *differently* across groups, so separate analyses were conducted in each sample. Thin-ideal internalization and pressures for thinness were significant positive predictors of eating pathology in both US and Iranian women.

**Conclusion:**

Both pressures for thinness and thin-ideal internalization appear to be relevant to eating pathology in women from both cultures. However, there may be important cross-cultural differences in the interpretation or experience of these constructs. Further understanding of this measurement non-invariance and the ways in which Iranian women may be uniquely impacted by Western values of appearance is a critical next step.

## Introduction

Despite evidence that some eating disorders (e.g., anorexia nervosa) may be culture-bound, data from the past few decades suggest that rates of eating pathology are increasing in women from non-Western societies (Miller and Pumariega, 2001; Keel and Klump, 2003; Makino et al., 2004; Jung and Forbes, 2007; Jung et al., 2009; Nakai et al., 2014; Pike and Dunne, 2015; Levinson and Brosof, 2016). In particular, research from Asia (e.g., China, Japan; Makino et al., 2006; Jung and Forbes, 2007; Jung et al., 2009; Nakai et al., 2014; Levinson and Brosof, 2016) and the Middle East (i.e., Iran, Jordan, Kuwait, Qatar; Musaiger et al., 2016; Al-Kloub et al., 2018; Al-Thani and Khaled, 2018; Sahlan et al., 2020c, 2021a,b) shows evidence of eating pathology globally. In fact, some research suggests that levels of eating pathology symptoms (e.g., drive for thinness, binge-purge behaviors, excessive exercise) among women in non-Western societies are comparable (Jung and Forbes, 2007; Forbes and Jung, 2008; Jung et al., 2009; Sahlan et al., 2021c) or even greater than samples in the United States (US; Davis and Katzman, 1998; Jung and Forbes, 2007; Forbes and Jung, 2008; Jung et al., 2009). These data highlight the need to further explore the etiology of eating pathology in non-Western cultures.

One of the strongest predictors of eating pathology in women from Western cultures is exposure to and internalization of Western values and ideals of appearance (Keel and Forney, 2013; Culbert et al., 2015). According to the Tripartite Model (Thompson et al., 1999; van den Berg et al., 2002), sociocultural idealization of thinness in women increases perceived pressures from the media, family, and peers to become thin and heightens *thin-ideal internalization* (i.e., endorsement of thinness as paramount to one’s beauty and value; Thompson et al., 1999; van den Berg et al., 2002; Fitzsimmons-Craft, 2011; Keel and Forney, 2013). Increasing rates of eating pathology in non-Western women could arise from societal and cultural changes, including rapid economic growth, disintegration of extended family network, increased access to technology, and urbanization (Miller and Pumariega, 2001; Keel and Klump, 2003; Makino et al., 2004; Pike and Dunne, 2015). Indeed, these cultural and societal changes can result in increased exposure to Western values about physical beauty (Miller and Pumariega, 2001). For example, studies of Fijian women linked exposure to mainstream Western media through the introduction of television with increased eating pathology (Becker et al., 2002, 2011; Becker, 2004) as well as endorsement of desire for thinness as a means to increase resemblance to girls/women portrayed on television (Becker et al., 2002). Thus, examination of sociocultural factors for eating pathology (i.e., thin-ideal internalization; pressures for thinness) is a critical next step in elucidating increases in eating pathology among non-Western cultural groups (Jung and Forbes, 2007; Forbes and Jung, 2008; Gan et al., 2011; Chang et al., 2013; Jackson et al., 2016; Shin et al., 2017).

One understudied group that may have unique protective or risk factors for body image and eating pathology is Iranian college women. Broadly, factors associated with modest dress have been linked with sociocultural factors, particularly among populations in which modest dress is strongly encouraged, such as hijab-wearing Muslim women. For example, findings among Muslim women in the US (Dunkel et al., 2010), France (Kertechian and Swami, 2016), England (Swami et al., 2014), Australia (Mussap, 2009), and Serbia (Durovic et al., 2016) suggest that women who wore the hijab endorsed lower pressures for thinness, thin-ideal internalization, and body image concerns, compared to women who did not. Additionally, hijab wearing was associated with positive body image among Muslim Israeli women (though uncorrelated with pressure for thinness or thin-ideal internalization; Sidi et al., 2020). These findings suggest that modest dress (e.g., wearing the hijab) may be protective against eating pathology. One problem with existing findings on hijab wearing Muslim women is that most research has been conducted in communities where wearing a hijab is *voluntary* and Muslims are a minority. Importantly, the above-mentioned studies did not examine measurement across Muslim and non-Muslim women to indicate whether the scales would perform similarly across groups.

Consistent with objectification theory (e.g., body regarded as an object, not part of a person) (Fredrickson and Roberts, 1997; Muehlenkamp et al., 2005), women in Western media are often objectified and shown in sexualized poses with limited clothing (Fredrickson and Roberts, 1997; Muehlenkamp et al., 2005). This body objectification is in stark contrast with Islamic value for modest dress, such as hijab-wearing in Muslim women. Iranian culture is particularly unique because modest dress (i.e., wearing Islamic-head and -body coverings like a *hijab* and a *manteau*) has been *legally* mandated for women since the 1979 Iranian revolution and mainstream Western media and/or Western media exposure has been largely censored. As such, the attitudes towards dress, modesty, and Western media and/or Western media exposure may be different among women in Iran, in comparison to women from Westernized cultures and cultures in which Muslim women use dress style *voluntary*. Nonetheless, emerging findings suggest that mainstream Western media and/or Western media exposure may extend to Iran (Shahyad et al., 2018; Sahlan et al., 2020a), possibly explaining the increasing incidence of eating pathology among Iranian women (Sahlan et al., 2020c, 2021a,b), but findings have been mixed. For example, data show positive associations among (1) thin-ideal internalization and body image concerns among adolescent Iranian girls (Shahyad et al., 2018) and (2) thin-ideal internalization, pressure for thinness, and body dissatisfaction in Iranian college women (Sahlan et al., 2020a). In contrast, research using community samples of Iranian women suggest that (1) hijab does not decrease body image concerns (Pahlevan Sharif et al., 2019); (2) family/peer pressures for thinness and Western social media are not predictors for eating pathology (Garrusi and Baneshi, 2013); and (3) conservative hijab-wearing (i.e., loose-fitting dress or chador) Iranian women who attend health clubs reported lower body dissatisfaction than women wearing least conservative hijab (i.e., short scarf, possibly leaving some hair uncovered; Rastmanesh et al., 2009).

One possible explanation for the discrepancies between college and community Iranian samples may be generational/subcultural differences in religiosity, which can protect against body image concerns in community samples of women (Pahlevan Sharif et al., 2019). Research suggests that sociocultural factors such as thin-ideal internalization and pressures for thinness are common among college women which in turn are related to eating pathology (i.e., Fitzsimmons-Craft, 2011). Relatedly, college women generally report higher eating pathology than community samples, which may demonstrate the importance of sociocultural factors among college women in Iran (i.e., Garrusi and Baneshi, 2013; Sahlan et al., 2020c).

Furthermore, exposure to Western media (especially *via* uncensored social media, such as Instagram; Sharifi et al., 2016) may be particularly salient for college populations compared to community samples (Garrusi and Baneshi, 2013). These findings could reflect increased Westernization of beauty standards, among younger and/or more educated Iranians. Indeed, recent findings among Iranian college women show: (1) associations between eating pathology and typical Western patterns of social comparison (i.e., body, eating, exercise; Sahlan et al., 2020b, 2021c); and, (2) emergence of desire for weight loss as the most salient contributor to eating pathology (Sahlan et al., 2021c). Thus, Western-based sociocultural theories of eating pathology development, such as the Tripartite Model (e.g., Thompson et al., 1999; Stice, 2001; Pennesi and Wade, 2016; Schaefer and Thompson, 2018; Weissman, 2019) are likely salient to Iranian women, despite possible cross-cultural differences from Western samples. Accordingly, determining whether measures of sociocultural factors (i.e., pressures for thinness; thin-ideal internalization) capture similar constructs in Iranian and US women and evaluating the extent to which these factors contribute to eating pathology in Iranian and US college women is a critical next step.

This study examined sociocultural factors and eating pathology in Iranian and US college women. Given that compulsory Islamic dress and Western media censorship is normative in Iran, we were interested in examining whether Iranian women would demonstrate 1) lower mean levels of eating pathology and sociocultural factors and 2) weaker associations between sociocultural factors and eating pathology than US women. This was also the first known study to test measurement invariance between English and Farsi visions of the EDE-Q; thus, before making comparisons between US and Iranian participants, we aimed to determine measurement invariance across the English and Farsi versions of the scale (Fischer and Karl, 2019). If the items/constructs were relatively invariant across groups, we could make meaningful comparisons between US and Iranian women. If cross-cultural differences in the item(s) or constructs emerged, we could not meaningfully make between-group comparisons and would need to limit our analyses to within-group approaches. However, in that case, the information would contribute to the literature by suggesting that Iranian women do not interpret these scales/constructs in a conceptually similar manner to US women.

## Materials and methods

### Participants

Participants were 1,040 college women from a large university in the Southwestern US (*n* = 709) and two large universities in Iran (*n* = 331). Data from these samples have appeared in two independent studies examining eating pathology and sociocultural factors in women from the US (Rakhkovskaya and Warren, 2016; collected 2013–2014) and Iran (Sahlan et al., 2019, 2020a; collected 2015–2017).

The US sample contains a subset of women from the original study. Some participants (*n* = 306) were excluded from the current study because they did not complete one of the primary measures for this study (i.e., the SATAQ-4) due to a survey error. Additionally, 79 US participants older than 23 years (or of unknown age) were excluded to more closely match the demographics of the Iranian dataset. These exclusions resulted in a final sample size of 709 US participants. Notably, the US sample was ethnically and racially diverse (28% Latina, 26% European American; 25% Asian American; 13% African American; 9% other races; see Table 1), consistent with the university student population (e.g., designated Minority Serving Institution).

**Table 1:** Sample descriptives by country.

	US *n* = 709	Iran *n* = 331	*t*	
Age _M ± SD (Range)_	19.04 ± 1.38 (18–23)	20.11 ± 1.32 (18–23)	12.03***	
BMI _M ± SD (Range)_	23.42 ± 4.71 (15.35–51.68)	21.64 ± 3.28 (15.42–32.03)	6.19***	
Perceived weight				
Underweight n (%)	63 (8.9%)	59 (17.8%)		
Normal weight n (%)	453 (63.9%)	231 (69.8%)		
Overweight n (%)	123 (17.3%)	36 (10.9%)		
Obese n (%)	65 (9.2%)	5 (1.5%)		
Race/Ethnicity n (%)				
Latina	201 (28.3%)	–		
European American	181 (25.5%)	–		
Asian American	175 (24.7%)	–		
African American	91 (12.8%)	–		
Other races	61 (8.6%)	–		
SATAQ-4				
Thin-ideal internalization _M ± SD (Range)_	3.50 ± 0.85 (1.0–5.0)	2.75 ± 0.98 (1.0–5.0)		
Pressures for thinness _M ± SD (Range)_	2.91 ± 1.0 (1.0–5.0)	2.04 ± 0.92 (1.0–4.92)		
EDE-Q				
Eating pathology global score _M ± SD (Range)_	2.07 ± 1.32 (0.05–5.48)	1.52 ± 1.27 (0.0–5.81)		
*n* (%) above clinical cut off^a^	60 (8.5%)	18 (5.4%)		

SATAQ-4, Sociocultural Attitude Toward to Appearance Questionnaire-4. EDE-Q, Eating Disorder Examination Questionnaire. ^a^ We used ≥ 4.0 as a clinical cutoff point (i.e., Luce and Crowther, 1999). *** *p* < 0.001.

### Procedure

All data collection was approved by the respective university ethics boards and informed consent procedures were followed. US participants completed questionnaires in English, through a secure survey platform online (i.e., Qualtrics). Iranian participants completed paper questionnaires in Farsi, in person.

### Measures

#### Demographic information

Participants completed questions regarding age, gender, height and weight (used to calculate Body Mass Index [BMI, kg/m^2^]). Despite limitations of self-reported height/weight data, self-reported body weight is generally highly correlated with actual weights (e.g., *r’*s ~ 0.90; Kuczmarski et al., 2001; Olfert et al., 2018).

#### Sociocultural attitude toward appearance questionnaire-4

The thin-ideal internalization, as well as the family, peer, and media pressures for thinness subscales of the SATAQ-4 (Schaefer et al., 2015) assessed endorsement of mainstream Western ideals of physical appearance. SATAQ-4 items are rated on a 5-point Likert-type scale from 1 (*Completely disagree*) to 5 (*Completely agree*) with higher scores indicating greater endorsement of Western ideals. Psychometric evaluations of English and Farsi versions of the SATAQ-4 showed strong psychometric properties (i.e., factor structure, convergent validity, internal consistency; Schaefer et al., 2015; Sahlan et al., 2019, 2020a). Additionally, the SATAQ-4 was invariant across Black and White college women in the US (Burnette et al., 2020). Family, media, and peer pressures for thinness were highly positively correlated with pressures for thinness subscale (*r*’s 0.75–0.84, *p*’s < 0.001 for all); thus, similar to prior studies (e.g., Rohde et al., 2015; Rakhkovskaya and Warren, 2016; Habashy and Culbert, 2019) an averaged pressures for thinness score was used in analyses. In the present study, Cronbach’s α among US and Iranian college women were 0.76–0.81 (Thin-Ideal Internalization) and 0.91–0.92 (Pressures for Thinness), respectively.

#### Eating disorder examination questionnaire

The EDE-Q (Fairburn and Beglin, 2008) assesses eating disorder symptoms over the past 28 days, including dietary restraint, weight, shape, and eating concerns. Items are rated on a 7-point scale ranging from 0 (*No days*) to 6 (*Every day*) so higher summed scores indicate greater eating pathology. The factor structure of the scale was problematic in the literature (i.e., Rand-Giovannetti et al., 2020), but findings suggest that the EDE-Q has strong discriminant and convergent validity, and internal consistency in English and Farsi versions (Luce et al., 2008; Lavender et al., 2010; Sahlan et al., 2021a,b). In the present study, Cronbach’s α was.93 (Global Score) in both US and Iranian samples.

### Data preparation

Scales containing 10 or more items were prorated for participants missing 10% or fewer of items. Scores were coded as missing for participants missing items on scales that contain less than 10 items. Among US women, data missingness ranged from 0.1 to 0.6% (*n* = 1–4) for SATAQ-4 scales, 2.7–3.8% (*n* = 19–27) for EDE-Q scales, and 0.7% (*n* = 5) for weight/height. A small proportion of Iranian women 0.9% (*n* = 3) were missing EDE-Q data. All missing data were handled using pairwise deletion. BMI and age were log-transformed in analyses to adjust for skewness. Standardized scores were used in analyses to ease interpretation.

### Data analysis

We used *R Studio* to test for measurement invariance and IBM SPSS 25 for all other statistical analyses. Measurement invariance analyses of the SATAQ-4 and EDE-Q were conducted by country of origin. A succession of nested multi-group analysis models was run, and fit was compared between models, per recommendations (Byrne, 2016). Specifically, with regard to multi-group analysis, we examined four models: unconstrained model (i.e., configural invariance; Model 1); measurement weights (i.e., metric invariance; Model 2); structural means and structural covariances (i.e., scalar equivalence; Models 3 and 4, respectively). The change of chi-squared statistics was used for model comparisons, such that the least restrictive models were subtracted from the most restrictive models. In other words, a *non-significant Δ*χ^2^ indicated that the *least* restrictive model has better fit.

If the scales showed measurement invariance, we planned to proceed with testing mean differences between US and Iranian participants on thin-ideal internalization, pressures for thinness, and eating pathology and to explore whether the magnitude of the relationship between pressures for thinness or thin-ideal internalization and eating pathology differed between groups. In the absence of establishing this measurement equivalence, we would proceed with a multi-group approach–descriptive statistics and regression models testing whether pressures for thinness and/or thin-ideal internalization predicted eating pathology would be conducted separately in each sample.

## Results

### Measurement invariance

Tests showed both the SATAQ-4 and the EDE-Q as non-invariant between Iranian and US women, counter-indicating direct between-group comparisons (see Table 2). Specifically, multi-group analysis confirmed configural invariance of the SATAQ-4 by country of origin (i.e., model structure was confirmed for both US and Iranian participants; Model 1), but neither metric nor scalar invariance were confirmed. SATAQ-4 factor loadings, intercepts, and covariances were non-equivalent for US and Iranian participants, as evidenced by a significant worsening of fit from Model 1 to Models 2, 3, and 4, respectively.

**Table 2:** Measurement invariance results by country.

Model	WLSMV χ^2^ (df)	*Δ*χ^2^ (df)	RMSEA	CFI
1. Assuming unconstrained model to be correct	540.68 (398)	–	0.05	0.93
2. Assuming measurement weights to be correct	712.58 (415)	42.84 (17)***	0.06	0.92
3. Assuming structural means to be correct	1062.55 (432)	366.95 (17)***	0.07	0.87
4. Assuming structural covariances to be correct	3063.40 (437)	330.82 (5)***	0.12	0.60

WLSMV, Weighted Least Squares With Mean and Variance Adjustment; *χ*^2^, chi-squared statistic; *df*, degrees of freedom; ∆*χ*^2^, chi-squared difference statistic; RMSEA, root-mean-square error of approximation; CFI, comparative fit index; ***p < 0.001; for model comparisons, least restrictive models are subtracted from most restrictive models. A non-significant ∆χ^2^ indicates that the least restrictive model has better fit, or in other words, a significant χ^2^ indicates more restrictive models were not at least as fitting.

On the EDE-Q, models did not converge after 100,000 iterations, indicating the model structures were configurally non-invariant (thereby also indicating metric and scalar non-invariance). Factor analyses (see Supplementary Tables 2, 3) indicated that, while the English and Farsi EDE-Q’s both had 4-factor structures, the compositions of each respective factor differed substantially in content. As such, it was not possible to identify and drop non-invariant items from the EDE-Q to attain non-invariance between the English and Farsi versions. Descriptive statistics and subsequent analyses were conducted separately for Iranian and US women.

### Descriptive statistics

US and Iranian women endorsed a range of scores on the pressures for thinness, thin-ideal internalization, and eating pathology scales (See Table 1). Incidence of clinical-level eating pathology (i.e., > 4.0 EDE-Q cut-off; Luce and Crowther, 1999) was notable in both samples, but higher in US women (8.5%) than Iranian women (5.4%). Pearson and partial correlations showed significant positive associations among thin-ideal internalization, pressures for thinness, and eating pathology in both samples, respectively (see Supplementary Table 1). Furthermore, Iranian women were significantly older (*t* = 12.03, *df* = 1,038, *p* < 0.001; Table 1) and had lower BMI (*t* = 6.19, *df* = 1,033, *p* < 0.001) than US women. Some significant intercorrelations were found between age, BMI, pressures for thinness, thin-ideal internalization, and eating pathology (see Supplementary Table 1); thus, age and BMI were included as covariates in regression analyses.

With respect to the racial/ethnic diversity of the US sample, we also tested the magnitude of correlations among study variables, with and without controlling for race/ethnicity. Partial correlations showed very minimal differences (see Supplementary Table 1) so primary analyses did not adjust for race/ethnicity in US women.

### Regression models

Table 3 shows results of linear regression, separately for US and Iranian participants. Thin-ideal internalization and pressures for thinness were both significant positive predictors of eating pathology in both samples, even after adjusting for age and BMI. These regression models explained 52 and 46% of disordered eating variance in US and Iranian women, respectively.

**Table 3:** Regression models predicting eating pathology.

	*Β*	*t*	*p*	*R^2^*
US women				
Age	−0.03	−0.96	0.34	0.52
BMI	0.22	7.36	<0.01	
Pressure for thinness	0.35	10.84	<0.01	
Thin-ideal internalization	0.42	14.01	<0.01	
Iranian women				
Age	0.02	0.52	0.61	0.46
BMI	0.36	7.82	<0.01	
Pressure for thinness	0.26	5.31	<0.01	
Thin-ideal internalization	0.26	5.53	<0.01	

*β*, standardized beta coefficient; *t*, *t*-value; *p*, value of *p*; *R*^2^, percentage of disordered eating variance explained by model.

## Discussion

Consistent with some previous research in Western (Knauss et al., 2009; Francisco et al., 2015; Llorente et al., 2015; Schaefer et al., 2015; Habashy and Culbert, 2019) and non-Western (Mellor et al., 2009; Gan et al., 2011; Chang et al., 2013; Jackson and Chen, 2015; Shin et al., 2017) societies, we found that pressures for thinness and thin-ideal internalization positively predicted eating pathology in both samples. These findings indicate that Western sociocultural factors extend to non-Western societies, such as Iran. Furthermore, although the proportion of women scoring above the EDE-Q clinical threshold was higher in US women, a notable proportion of Iranian (5.4%) women also demonstrated clinical levels of eating pathology using this cutoff. In sum, thin-ideal internalization and perceived pressures for thinness are associated with eating pathology in both US and Iranian women, yet there may be cross-cultural differences in the interpretation or experience of these constructs. Gaining an understanding of the measurement non-invariance and how Iranian women may be uniquely impacted by Westernized appearance attitudes and eating pathology will be critical for future cross-cultural work. Indeed, the sociocultural context in which US and Iranian college women live is different, which in turn, could affect the ways in which women experience appearance-based factors in these cultures.

Our results tentatively suggest that sociocultural appearance-based factors may be salient for both US and Iranian women and predictive of eating pathology outcomes. These findings could be interpreted to suggest that the hijab is no longer a protective factor against eating pathology and aligned with a previous study in Iran (Pahlevan Sharif et al., 2019). The moderate endorsement of pressures for thinness and thin-ideal internalization among Iranian women in this study also fits with prior work suggesting that Iranian women are increasingly becoming more westernized by adapting Western beauty standards (Sahlan et al., 2021c) and that Western-based sociocultural theories of eating pathology extend to Iranian college women (Sahlan et al., 2021c). These findings may also generalize to other Middle Eastern women, as evidenced by findings of recent shift from a larger to a thin body ideal (Eapen et al., 2006; Eshak et al., 2020). Anecdotally, despite official Western media censorship, Iranians demonstrate increased familiarity with Western media, *via* the international Dish television network and/or online social media (e.g., Instagram; Sharifi et al., 2016). Changes in media consumption may have shifted the standards of appearance (Sharifi et al., 2016), as well as increased tendency towards body image-related social comparison (Sahlan et al., 2020b, 2021c), particularly among Iranian college women (Sahlan et al., 2020c, 2022). Indeed, in contrast to findings among community Iranian women (Garrusi and Baneshi, 2013), pressures for thinness predicted eating pathology for this sample, consistent with prior research showing college women as a particularly high-risk group for Western sociocultural influences and eating pathology (Berg et al., 2009).

## Limitations and future directions

The findings of this study must be considered in light of its limitations. First, the cross-sectional design does not allow us making causal claims. Accordingly, future longitudinal studies should verify that increased thin-ideal internalization and pressures for thinness predicts the subsequent development of eating pathology in Iranian women. Second, due to the measurement non-invariance, we cannot verify whether the scales measured precisely the same constructs (i.e., pressures for thinness; thin-ideal internalization; eating pathology) across the two groups. Future studies need to conduct further cross-cultural research, as well as consider scale revision to potentially eliminate particularly non-invariant scale items and/or use different self-report or interview-based assessments.

Third, despite the anecdotally observed accessibility of mainstream Western media in Iran, we did not directly measure the extent of exposure to Western and local media in connection to eating pathology. As such, future studies should assess mainstream media access and exposure among Iranians, as well as an analysis of thin-ideal messages in Western vs. Iranian media. Fourth, the study included only college women. Therefore, as the results cannot be generalized to other demographic groups (e.g., college men, adolescents, middle-aged and older adults), all of whom should be investigated in the future. Further, we did not assess nature or type hijab-wearing in the US and Iranian samples and this may be important to examine in future work (Mussap, 2009; Rastmanesh et al., 2009). For example, Rastmanesh et al. (2009) found that Iranian women wearing the most conservative hijab (i.e., loose-fitting dress or chador) demonstrated lower body dissatisfaction than women wearing least conservative/secular hijab (i.e., short scarf, possibly leaving some hair uncovered). Finally, the US data was collected earlier (2013–2014) than Iranian data (2015–2017); however, it is unlikely that this timeframe difference unduly impacted our results, as the mean levels and intercorrelations between key study variables in our 2013–2014 US sample are similar to those found in other US college samples that were collected between 2015 and 2017 (e.g., Fitzsimmons-Craft et al., 2014; Schaefer et al., 2017; Habashy and Culbert, 2019).

## Data availability statement

The original contributions presented in the study are included in the article/Supplementary material, further inquiries can be directed to the corresponding author.

## Ethics statement

The studies involving human participants were reviewed and approved by Iran University of Medical Sciences and University of Nevada, Las Vegas. The patients/participants provided their written informed consent to participate in this study.

## Author contributions

RS: collection of Iran data, data analysis, and lead role on writing original draft and editing the manuscript. LA: collection of US data, data analysis, and writing/editing the manuscript. JH and KC: writing/editing the manuscript. CW: supervision of US data collection and writing/editing the manuscript. All authors contributed to the article and approved the submitted version.

## Conflict of interest

CW was employed by the company Choose Honesty, LLC, Las Vegas, NV, United States.

The remaining authors declare that the research was conducted in the absence of any commercial or financial relationships that could be construed as a potential conflict of interest.

## Publisher’s note

All claims expressed in this article are solely those of the authors and do not necessarily represent those of their affiliated organizations, or those of the publisher, the editors and the reviewers. Any product that may be evaluated in this article, or claim that may be made by its manufacturer, is not guaranteed or endorsed by the publisher.
