# Supplementary material for: Sociocultural correlates of eating pathology in college women from US and Iran
Source: Front Psychol. 2022 Sep 28;13:966810. doi: 10.3389/fpsyg.2022.966810 (PMC9554628; doi:10.3389/fpsyg.2022.966810)
Supplement: Supplementary file 1 [file Table_1.DOCX]

**Supplementary Table 1**

*Pearson and Partial Correlations between Sociocultural Factors and Eating Pathology in US and Iranian Women.*

|  | Thin-ideal internalization | Pressures for thinness | Eating Pathology |
| --- | --- | --- | --- |
| Thin-ideal internalization | - | .42*** (.46, .47)*** | .57*** (.60, .60)*** |
| Pressures for thinness | .43*** (.32)*** | - | .60*** (.55, .55)*** |
| Eating pathology | .49*** (.38)*** | .52*** (.36)*** | - |

*Note*. Bivariate Pearson correlations for the US sample are above the diagonal; Iranian results are below the diagonal. Partial correlations are shown in parentheses; partial correlations in the Iranian sample controlled for BMI and age only, whereas partial correlations in the US sample controlled for BMI and age only (first coefficient in parentheses) or BMI, age, and race/ethnicity (second coefficient in parentheses). Pressures for thinness is a composite score of family, peers, and media pressures from the Sociocultural Attitudes Towards Appearance Quesitonnaire-4 (SATAQ-4). Eating pathology = Eating Disorder Examination Questionnaire (EDE-Q) global score. *** = *p* < .001.

| **Supplementary Table 2**  *Exploratory Factor Analysis of the EDE-Q in US Women.* | | | | |
| --- | --- | --- | --- | --- |
|  | F1 | F2 | F3 | F4 |
| EDEQ1 | -.00 | **.77** | -.04 | .12 |
| EDEQ2 | .08 | *.41* | *.38* | -.19 |
| EDEQ3 | -.02 | **.84** | -.07 | .05 |
| EDEQ4 | -.09 | **.79** | .02 | .03 |
| EDEQ5 | .10 | *.37* | *.36* | -.04 |
| EDEQ6 | -.03 | .14 | -.02 | **.62** |
| EDEQ7 | -.05 | -.00 | **.82** | -.05 |
| EDEQ8 | -.04 | -.04 | **.88** | .06 |
| EDEQ9 | -.04 | -.00 | *.48* | *.31* |
| EDEQ10 | -.05 | -.04 | .20 | **.75** |
| EDEQ11 | .22 | -.01 | -.00 | **.68** |
| EDEQ12 | .04 | .115 | -.08 | **.81** |
| EDEQ19 | .22 | -.10 | **.44** | .03 |
| EDEQ20 | **.76** | .06 | .02 | .02 |
| EDEQ21 | **.76** | .03 | .05 | .00 |
| EDEQ22 | **.73** | -.04 | .09 | -.10 |
| EDEQ23 | **.75** | .01 | -.03 | .17 |
| EDEQ24 | **.76** | .01 | -.02 | .17 |
| EDEQ25 | **.85** | -.01 | -.00 | .05 |
| EDEQ26 | **.86** | -.01 | -.04 | .05 |
| EDEQ27 | -.16 | *.05* | -.06 | -.02 |
| EDEQ28 | *.13* | -.02 | -.03 | -.06 |
| *Note*. Factor loading obtained using principal axis factoring with promax rotation. Factor loading ≥.44 in boldface. Low and Cross-loading factors are in italic. | | | | |

| **Supplementary Table 3**  *Exploratory Factor Analysis of the EDE-Q in Iranian Women.* | | | | | |
| --- | --- | --- | --- | --- | --- |
|  | F1 | F2 | F3 | F4 | F5 |
| EDEQ1 | *.40* | .03 | *.60* | -.03 | -.18 |
| EDEQ2 | -.17 | .05 | **.87** | .05 | -.14 |
| EDEQ3 | .22 | -.04 | **.64** | -.11 | .02 |
| EDEQ4 | .05 | .05 | **.71** | -.17 | -.04 |
| EDEQ5 | .23 | -.01 | **.41** | -.00 | .17 |
| EDEQ6 | **.52** | -.16 | .14 | .16 | -.08 |
| EDEQ7 | -.15 | .01 | **.42** | .16 | .25 |
| EDEQ8 | .00 | -.02 | *.39* | .18 | .25 |
| EDEQ9 | **.50** | -.15 | .22 | .16 | .17 |
| EDEQ10 | **.95** | -.11 | -.05 | .10 | -.03 |
| EDEQ11 | **.83** | .23 | -.08 | -.09 | -.01 |
| EDEQ12 | **.85** | .17 | .03 | -.10 | -.09 |
| EDEQ19 | -.04 | -.01 | -.12 | -.01 | **.76** |
| EDEQ20 | **.65** | .07 | -.11 | .00 | .21 |
| EDEQ21 | .04 | .08 | .00 | -.13 | **.73** |
| EDEQ22 | .02 | .13 | -.03 | **.88** | -.09 |
| EDEQ23 | .07 | .03 | -.06 | **.85** | -.03 |
| EDEQ24 | .01 | *.38* | .05 | .06 | .21 |
| EDEQ25 | .08 | **.79** | -.07 | .07 | -.03 |
| EDEQ26 | .04 | **.87** | -.03 | .02 | -.04 |
| EDEQ27 | -.06 | **.90** | .09 | .00 | -.00 |
| EDEQ28 | -.06 | **.74** | .09 | .03 | .09 |
| *Note*. Factor loading obtained using principal axis factoring with promax rotation. Factor loading ≥.41 in boldface. Low and Cross-loading factors are in italic. | | | | | |
